# Efficacy of *Oryza sativa* husk and *Quercus phillyraeoides* extracts for the *in vitro* and *in vivo* control of fungal rot disease of white yam (*Dioscorea rotundata* Poir)

**DOI:** 10.1186/2193-1801-3-711

**Published:** 2014-12-03

**Authors:** Victor Ohileobo Dania, Olubunmi Omowunmi Fadina, Maria Ayodele, P Lava Kumar

**Affiliations:** International Institute of Tropical Agriculture (IITA), Ibadan, Nigeria; Department of Crop Protection and Environmental Biology, University of Ibadan, Ibadan, Nigeria

**Keywords:** *Dioscorea rotundata*, Agroecological zones, Pathogens, Biopesticide, Post-harvest rot

## Abstract

Tuber rot disease is a major constraint to white yam (*Dioscorea rotundata*) production, accounting for 50-60% of annual yield losses in Nigeria. The main method of control using synthetic fungicides is being discouraged due to human and environmental health hazards. The potential of *Oryza sativa* husk (OSH) and *Quercus phillyraeoides* (QP) extracts for the *in vitro* and *in vivo* control of six virulent rot-causing fungal pathogens, *Lasiodiplodia theobromae, Aspergillus niger, Rhizoctonia solani, Penicillium oxalicum, Sclerotium rolfsii*, and *Fusarium oxysporum* was evaluated, using five different extract concentrations of 0.5%, 1.0%, 1.5%, 2.5%, and 3.5% w/v. These fungi were isolated from rotted tubers of *D. rotundata*, across three agroecological zones in Nigeria―the Humid rainforest, Derived savanna, and southern Guinea savanna. All treatments were subjected to three methods of inoculation 48 hours before the application of both extracts and stored at 28 ± 2°C for 6 months. Radial mycelial growth of the test pathogens was effectively inhibited at concentrations ≤ 3.5% w/v *in vitro* for both OSH and QP extracts. Rotting was significantly reduced (P ≤ 0.05) to between 0 to 18.8% and 0% to 20.9% for OSH and QP extracts respectively. The extracts significantly (P ≤ 0.05) inhibited percent rot of the test pathogens at 3.5% concentration w/v *in vivo*. Rot incidence was, however, lower in replicate tubers that were inoculated, treated with extracts and exposed than treatments that were covered. Phytochemical analysis of OSH and QP extracts revealed the presence of secondary metabolites such as alkaloids, flavonoids, saponins, tannins, ferulic acid, phlobatanins, Terpenoids, phenols, anthraquinone and pyroligneous acid. The efficacy of both extracts in reducing rot in this study recommends their development as prospective biopesticide formulation and use in the management of post-harvest rot of yam tubers.

## Introduction

Yam is mainly cultivated as a staple crop across the humid and semi-humid tropics, especially in West Africa which accounts for about 90% of world yam production (Aboagyne-Nuamah et al. 2005). There are over 150 species of yam grown throughout the world (Purseglove 1985). White yam (*D. rotundata*) is the most predominant yam species and it constitutes about 80% of total yam produced worldwide [(Titteh and Saakwa 1994; Ejechi and Souzey 1999). Although the main constituent of the yam tuber is carbohydrate, it also contains an appreciable amount of proteins, vitamins and minerals (Babaleye 2003). It serves as an important source of income to many people. The tuber is of economic value and is the most important part of the crop. It can be eaten in different forms such as roasted, boiled, fried or better still made as pounded yam or as flour which is a delicacy in Nigeria. Yam is a very important and revered crop, especially in southern Nigeria where it has cultural importance exemplified by the annual new yam festival (Arua 1981; Okorji 1992). Water yam (*D. alata*) becomes an alternative only when the white yam is out of season or very costly.

Pests and diseases are the major limitation to cultivation; hence demand has always exceeded its supply in yam producting countries like Nigeria (Coursey 1983). Yam tubers are liable to infection by pathogens such as fungi, bacteria, viruses, and nematodes at various stages of its growth in the field. In most cases, infection starts in the field through injuries to the yam tubers and in transit to the market or barn for storage (Yoshida et al. 2000). Rot is an important factor affecting post-harvest shelf life of yams and losses can be very high. Losses of over 60% of *D. rotundata* tubers rot stored for six months have been reported (Ikotun 1983a, 1989). The yield loss ultimately reduces farmers’ income, available food supply and yam setts for the next growing season. (Amusa et al. 2003; Aidoo 2007). Besides the decrease in quantity, rot also reduces the quality of yam tubers thereby making them unattractive to prospective buyers. Yams are attacked by several diseases; fungi are, however, the major cause of post-harvest rot of yam tubers (Noon 1978; Dania 2012). Fungal pathogens such as *Lasiodiplodia theobromae*, *Aspergillus niger*, *Fusarium oxysporum*, *Rhizoctonia solani*, *Penicillium oxalicum*, and *Sclerotium rolfsii* are commonly associated with rotting of yam tubers (Ikotun 1989; Aidoo 2007).

Peasant farmers often quickly sell off their harvested yam tubers in order to avoid losses due to microbial decay. This action has adversely affected yam production, availability and utilization. Yam producers are always concerned with tuber quality from cultivation on the farm to storage in order to guarantee high price for their produce. The quality of yam setts for the next growing season is also very important to producers in order to ensure perpetuation of the crop and guarantee continuous supply. This, therefore, emphasizes the need for disease control.

Synthetic fungicides had been very popular in the control of agricultural pests and pathogens because of their ease of application, effectiveness and storage (Prakash and Rao 1994). However, in recent times, their use in crop protection programmes has come under increased criticisms worldwide for some obvious reasons. Their use results in adverse effects on the environment, persistence, pathogen resurgence due to resistance development, mammalian toxicity, elimination of beneficial soil micro and macro-organisms (GOAN 1999). There is, therefore, a need to search for cheap and eco-friendly control measures in the management of post-harvest rot of yam. Several authors have suggested the use of botanicals as an alternative to the hazardous effects of synthetic pesticides (Enikuomehin et al. 1998; Amadioha 2000; Hycenth 2008). However, most of the efforts with botanicals to control yam pathogens do not go beyond the *in vitro* trials. Hence this study investigated the potential of *Oryza sativa* husk and *Quercus phillyraeoides* extracts for the control of post-harvest fungal rot and extension of the shelf life of white yam.

## Materials and methods

### Collection and isolation of associated fungi from rotted yam tuber samples

Rotted tuber samples of *D. rotundata* were collected from different locations across three agroecological zones (AEZ): Humid rainforest, Derived savanna and southern Guinea savanna. Fifteen tubers showing rot symptoms were collected from fourteen locations across the three AEZ. A total of 210 rotten tubers were collected and evaluated for fungal rot pathogens. Each rotten sample was washed in running tap water and cut to expose the fresh necrotic tissues. From the areas in advance of the necrosis, tissue pieces were surface-sterilized with 10% sodium hypochlorite for 2 minutes, and rinsed in five changes of sterile distilled water (SDW) before plating on potato dextrose agar (PDA). The inoculated plates were incubated in the dark in a Gallenkamp incubator operating at a temperature of 28°C for 3 to 4 days and examined for fungal growth from the tissue pieces. Identification of the isolates was carried out using standard procedure (Barnett and Hunter 1998).

### Preparation of inoculum

Pure cultures of the pathogens were subcultured from different stock cultures. These were placed on PDA for 7–10 days depending on the pathogen, to allow full sporulation. This was then scooped out into a Warring blender containing one litre of SDW; a drop of Tween 20 (Polyoxyethylene sorbitan mono-oleat) detergent was added to aid the dislodgment of the spores from the medium. The spores were then strained off the mycelia fragments using a double layer of cheese cloth placed inside a sterile funnel. An aliquot of 0.1 mL spore concentration was then placed on an hemacytometer slide and the spores were quantified under a compound microscope. The final spore count and concentration was determined by using this method:

$$C_{1}V_{1}=C_{2}V_{2}$$

Where: C_1_ = Initial inoculum concentration, V_1_ = Initial volume of water used in streaking the culture plate

C_2_ = Final inoculum concentration desired, V_2_ = Final volume of water added to obtain desired concentration.

### Preparation of cold water extracts

Fresh *Oryza sativa* husks were washed thoroughly under running tap water and air-dried at a temperature of 28°C for 7–10 days until they became crisp. The dried plant husks were pulverized by blending using a high speed blender (Waring Commercial, Springfield, MO, USA) to form a fine powder while the bark of *Quercus phillyraeodes* (Oak plant) was dried in a Gallenkamp oven at 80°C for two days and crushed to a coarse powder in a mortar. Ultraviolet light (UV) was used to surface-sterilize the botanicals to prevent fungal and bacterial contaminants. Cold water extract was prepared by adding the powder to 100 mL of sterile distilled water in a 250- mL beaker. This was stirred vigorously and allowed to stand for 24 hours before filtering the extract through folds of sterile cheese cloth. Five different extract concentrations were prepared by blending 0.5 g, 1.0 g, 1.5 g, 2.5 g, and 3.5 g of each plant part in 100 mL of sterile distilled water to produce 0.5%, 1.0%, 1.5%, 2.5%, and 3.5% extract concentrations, respectively.

### Measurement of mycelial growth inhibition

This involved creating a four equidistant section on each Petri dish by drawing two perpendicular lines at the reverse bottom of the plate, the point of intersection indicating the center of the plate. This was done before dispensing PDA into each of the plates. An aliquot of 1 mL each of the extracts was separately introduced into the Petri dish containing 15mL PDA. A 5-mm cork borer was used to inoculate a disc of the pathogen culture and placed on the medium containing extract just at the point of intersection of the two previously drawn lines at the bottom of the Petri dish in three replicates. Control experiments were set up without the addition of any plant material. Inhibitory effect of the extract was expressed as percentage inhibition and calculated using the formula:

$$\%\;\mathrm{Mycelial}\;\mathrm{inhibition}=\left[\left(XC-YT\right)/XC\right]\times 100$$

Where: XC = Average diameter of control

YT = Average diameter of fungal colony with treatment

### Determination of the efficacy of extracts for control of tuber rot

The experiment was a 2 × 3 × 6 factorial in a randomized complete block design with three replicates. Both extracts that had earlier proved effective in the *in vitro* inhibition of the pathogens were used for the trials against the virulent fungal pathogens. Clean, healthy tubers of yam genotype TDr 95–18544 were washed under running tap water to remove adhering soil and surface-borne microflora. Eighteen tubers were inoculated per pathogen with three replicates in two batches. The first batch of nine tubers consisting of three sets of tubers in each treatment, were separately scratched, wounded, and bored, respectively. The tubers were inoculated with the test pathogens, the surface area treated with 1 ml of the extract and then exposed. Similarly, the second batch of nine tubers was scratched, wounded, bored, prior to inoculation and treated with the extract and covered with black polyethylene sheets. For scratched treatments, the tuber periderm was removed with a scapel up to 0.1 cm; wounded tubers were cut to a depth of 0.5 cm. Bored treatments were inoculated to a depth of 1 cm, using a 5-mm cork borer. The tubers were inoculated with the test pathogens 48 h before the application of the extracts and stored at room temperature (28 ± 2°C) in the yam barn for 6 months. Tubers were inoculated with a spore concentration of 10^6^ conidia/mL of the test pathogen. Control tubers were scratched, bored, wounded, and dipped in SDW and inoculated with test pathogen only. Percent rot inhibition was determined using the method:

$$\mathrm{Rot}\;\mathrm{inhibition}=\frac{\%\;\mathrm{decay}\;\mathrm{in}\;\mathrm{control}-\%\;\mathrm{decay}\;\mathrm{in}\;\mathrm{treated}\;\mathrm{tuber}}{\%\;\mathrm{decay}\;\mathrm{in}\;\mathrm{control}}$$

### Phytochemical screening of plant extracts

Fresh samples of *O. sativa* husk and *Q. phillyraeoides* bark extracts were prepared as previously described and were concentrated using a rotary evaporator. Chemical tests were carried out on the extracts for the qualitative determination of phytochemical constituents using standard procedures (Sofowora 1993). Mayer’s and Draggendoff’s reagents were used for test and confirmation of the presence of alkaloids in the extracts. The formation of a cream colour and reddish brown precipitate with Mayer’s and Draggendoff’s reagent respectively was regarded as the confirmatory test for the presence of alkaloids. The amount of total phenolics in extracts was determined with the Folin-Ciocalteu reagent. Gallic acid was used as a standard and the total phenolics were expressed as mg/g gallic acid equivalents (GAE). The formation of blue colour upon reaction with Folin-Ciocalteu reagent was the confirmatory test for the presence of phenols in the plant extract samples. Similarly, the formation of a red precipitate when the extract was boiled with 1% aqueous hydrochloric acid indicated the presence of phlobatannins. Other bioactive constituents such as saponins, tanins, flavonoids, steroids, terpenoides, anthraquinones etc. were also determined using standard procedures.

### Data analysis

All numerical data were statistically analysed using generalized linear model (GLM) of SAS. Means were separated using Least Significant Difference Test (LSD) and standard error was determined where applicable at 5% level of significance.

## Results

### Mycelial growth inhibition of test pathogens *in vitro*

Mycelial growth inhibition of the test pathogens ranged between 0% to 39.2% for OSH and 0% to 46.6% for QP extracts at 0.5% w/v concentration (Table 1). However, both extracts effectively inhibited growth of the pathogens at 3.5% w/v concentration (>70% inhibition). The result also shows that OSH could not completely inhibit *A.niger in vitro* at concentrations ≤ 2.5% w/v. There was no significant difference (P > 0.05) in the control *of L. theobromae and A. niger* at 0.5% w/v concentration for both OSH and QP extracts. However, the efficacy of both extracts differed significantly (P ≤ 0.05) at concentrations ≥1.0% ≤ 2.5% w/v. There was no significant difference in the efficacy of OSH and QP against *S. rolfsii* and *F. oxysporum* at 3.5% w/v concentration. The minimum inhibitory concentration for both extracts varied between 0.5% to 1.5% w/v depending on the test pathogen and extract.

**Table 1:** **Effect of** ***Oryza sativa*** **husk and** ***Quercus phillyraeoides*** **extracts on the mycelial growth of six virulent fungi after 7 days**

		Mycelial growth inhibition				
	Extract	0.5	1.0	1.5	2.5	3.5
*L. theobromae*	OSH	0.0e	100a	100a	100a	100a
	QP	0.0e	0.0e	11.1e	62.2 cd	86.1bc
*A. niger*	OSH	5.6 cd	11.1cde	70.0bc	77.8c	100a
	QP	6.7fcd	22.3c	33.3d	61.7 cd	77.4c
*S. rolfsii*	OSH	12.2c	22.2c	55.9 cd	89.2b	100a
	QP	0.0e	17.3 cd	65.4c	100a	100a
*P. oxalicum*	OSH	23.1abc	24.4c	67.2c	83.1bc	100a
	QP	30.0bc	52.3abc	100a	100a	100a
*R. solani*	OSH	4.4 cd	100a	100a	100a	100a
	QP	0.0e	0.0e	16.4de	88.0b	93.8b
*F. oxysporum*	OSH	39.2b	63.2bc	100a	100a	100a
	QP	46.6a	70.6b	90.6b	100a	100a
Std deviation		16.2	36.0	32.8	14.6	7.3

Means with the same letter along a column are not significantly different (P = 0.05).

Legend: OSH = *Oryza sativa husk*, QP = *Quercus phillyraeoides.*

### Efficacy of extracts for control of tuber rot

Rot development was significantly reduced (P ≤ 0.05) in tubers of *D. rotundata* inoculated with six virulent fungal pathogens 48 h before the application of *O. sativa* husk extract (Table 2). Rot initiation in replicate tubers that were scratched, inoculated with *R. solani, P. oxalicum* and *F. oxysporum* and treated with *O.sativ* a extract was completely inhibited. Rot inhibition was more effective in inoculated tubers that were either scratched or wounded and treated with the extracts than the bored treatments. However, the extract was more effective in tubers that were bored than those that were wounded, inoculated with *L. theobromae* and treated with the extract where rot was reduced to 17.4% and 18.8% respectively. Comparatively, rot was very pronounced in control treatments that were either wounded or bored and inoculated with the same pathogen but without extract application (52.2% to 78.2% rot). There was significant difference (P ≤ 0.05) in rot incidence between all the extract applied treatments and the control tubers that were inoculated with the test pathogens only.

**Table 2:** **Rot inhibition in tubers inoculated with six virulent fungi 48 h before the application of** ***O. sativa*** extract **stored for 6 months**

	Tuber rot (%)					
Fungus	Inoculated and exposed			Inoculated and covered		
	Scratch	Wound	Bore	Scratch	Wound	Bore
*L. theobromae*	5.5bc	10.4 cd	12.0d	18.8de	18.8d	17.4d
Control	12.8a	15.8bc	33.7bc	17.6bc	52.2a	78.2b
*R. solani*	0.0 cd	5.6de	11.8d	7.9e	11.2de	15.9de
Control	10.2b	9.5 cd	22.4bc	10.2de	49.4b	35.6 cd
*A. niger*	9.1b	12.7d	14.7 cd	12.1c	14.5d	18.8d
Control	12.3a	18.9b	34.3bc	19.7b	47.8b	82.7a
*P. oxalicum*	0.0 cd	7.5d	10.6d	12.3c	14.1d	13.2e
Control	6.1bc	10.2 cd	27.1d	22.4a	44.0bc	65.9abc
*S. rolfsii*	3.9c	7.1d	8.3de	8.5e	9.8e	13.0e
Control	8.8b	13.5c	39.3b	14.8abc	28.3 cd	73.7bc
*F. oxysporum*	0.0 cd	3.4e	8.7de	8.6e	9.1e	15.4de
Control	5.4bc	21.9a	44.7a	15.9abc	38.7c	53.7c
Std deviation	4.6	5.5	13.1	4.8	17.2	28.4

Means with the same letter along a column are not significantly different (P = 0.05).

Replicate tubers that were inoculated with *L. theobromae* and *R. solani* and treated with extracts differed significantly (P ≤ 0.05) except for treatments that were bored and exposed. Rot initiation also differed significantly (P ≤ 0.05) between treatments that were inoculated and exposed and those that were inoculated and covered. The extract was most effective in rot inhibition in tubers inoculated with *S. rolfsii* where rot was reduced to ≤ 13.0% and least effective in suppressing *A. niger* rot (≤ 18.8%). *Lasiodiplodia theobromae* and *A.niger* were most virulent in control tubers without extract application with 78.2% and 82.75% rot respectively.

*Quercus phillyraeoides* extract effectively inhibited rot in all the replicate treatments of *R. solani* that were scratched, inoculated and exposed (Table 3), but could not totally inhibit the pathogen rot in treatments that were wounded, bored and exposed or covered. However, the extract significantly (P ≤ 0.05) reduced rot of all the other pathogens relative to the control treatments in the *in vivo* trials. The extract was least effective against rot development in tubers that were bored, inoculated and either exposed or covered.

**Table 3:** **Rot inhibition in tubers inoculated with six virulent fungi 48 h before the application of** ***Q. phillyraeoides*** **extract stored for 6 months**

	Tuber rot (%)					
Fungus	Inoculated and exposed			Inoculated and covered		
	Sratch	Wound	Bore	Scratch	Wound	Bore
*L*. *theobromae*	9.9bc	14.0 cd	16.7bcd	13.8bc	17.1de	20.9de
Control	17.7a	29.8a	63.6a	19.5a	74.4a	69.8bc
*R. solani*	0.0e	11.5d	13.3d	10.5c	14.0cde	15.6e
Control	5.8 cd	16.5c	39.7abc	12.0bc	52.5 cd	45.2e
*A. niger*	7.5c	13.1 cd	19.6 cd	7.2 cd	20.6d	20.8de
Control	14.6b	25.6b	48.9bc	13.4bc	55.1c	78.3b
*P. oxalicum*	7.8c	10.7d	11.1de	8.3 cd	13.9cde	14.9e
Control	13.3b	22.3bc	32.4c	15.5b	48.2c	44.8 cd
*S. rolfsii*	4.7 cd	7.5e	11.3de	7.5 cd	17.3de	24.6d
Control	10.2bc	21.4bc	50.2b	19.8a	64.6b	81.7a
*F. oxysporum*	5.4 cd	11.5d	14.4d	10.5c	13.2cde	19.7de
Control	9.8bc	21.1c	47.6bc	16.6b	58.8bc	57.7abc
Std deviation	4.8	6.9	18.6	4.3	23.4	25.3

Means with the same letter along a column are not significantly different (P = 0.05).

The extract was most effective in rot suppression in tubers inoculated with *P.oxalicum* where rot was reduced to ≤ 14.9% and least effective in inhibiting *L. theobromae* (≤ 20.9% rot). *Sclerotium rolfsii* and *A. niger* were most virulent in control tubers with 81.7% and 78.3% rot respectively. There was significant difference in the control of rot development between the rot-causing fungi in tubers that were inoculated and exposed and those inoculated and covered (P ≤ 0.05). There was no significant difference in the effect of *Q. phillyraeoides* on rot initiation in treatments that were wounded and exposed (P > 0.05), except for tubers inoculated with *L. theobromae* and *A. niger* and *S. rolfsii* which showed significant difference (P ≤ 0.05). The efficacy of *Q. phillyraeoides* in the control of rot in tubers that were bored, inoculated and exposed differed significantly (P ≤ 0.05). Similarly, there was a significant difference among all treatments that were either wounded or bored and exposed (P ≤ 0.05). All treatments were significantly different from the control set up in all trials (P ≤ 0.05).

### Phytochemical screening of plant extracts

Phytochemical analysis of *Oryza sativa* husk and *Quercus phillyraeoides* extract revealed the presence of various active constituents (Table 4). Both extracts were found to contain alkaloids, flavonoids, saponins, tannins, terpenoids, phenol and anthraquinone. However, Phlobatanins and ferulic acid were lacking in *Q. phillyraeoides* extract. Similarly, pyroligneous acid was absent in Oryza sativa husk extract.

**Table 4:** **Phytochemical analysis of plant extracts**

Exract concentration mg/ml		
Phytochemical	***O. sativa***	***Q. phillyraeoides***
Alkaloids	2.16 ± 0.15	1.07 ± 0.03
Flavonoids	0.17 ± 0.20	0.187 ± 1.42
Saponins	3.30 ± 0.23	1.99 ± 0.02
Tannins	0.77 ± 0.11	0.17 ± 0.01
Phlobatanins	0.35 ± 0.01	0.00
Ferulic acid	5.69 ± 0.08	0.00
Terpenoids	0.71 ± 0.43	0.38 ± 0.05
Phenol	3.21 ± 0.04	2.59 ± 0.01
Anthraquinone	0.07 ± 0.01	0.45 ± 0.05
Pyroligneous acid	0.00	7.74 ± 0.12

Ferulic acid had the highest concentration of 5.69 mg/ml amongst the bioactive compounds contained in *Oryza sativa* husk extract, while pyroligneous acid had the highest concentration of 7.74 mg/ml in *Quercus phillyraeoides* extract.

## Discussion

Naturally, there are many plants for use in natural crop protection in order to ensure safe and healthy environment. These botanicals provide a viable alternative to synthetic pesticides because they are harmless to beneficial soil microorganisms such as symbiotic nitrogen-fixing bacteria, they are cheap and readily available, and they do not accumulate in the food chain. *Oryza sativa* extract used in this study completely inhibited the mycelial growth and conidia production in *A. niger, P. oxalicum*, and *F. oxysporum* at 3.5% w/v concentrations *in vitro* as revealed in Table 1. Comparatively, control plates showed distinct morphological features such as cluster of conidiospores borne in sterigmata and phaliades for *A. niger,* and *P. oxalicum*, respectively under microscopic examination. *Fusarium oxysporum* had spindle-shaped conidia. Similarly, they effectively inhibited the production of pycnidia in *L. theobromae* and the formation of sclerotia in *R. solani* and *S. rolfsii*. These results agree with the findings of Abiala 2008 who reported the potential of *O. sativa* husk extract for the *in vitro* control of the black sigatoka pathogen, *Mycosphaerella fijiensis* Also, Dhaliwal et al. (1993) found extracts from several rice varieties to be effective in inhibiting *R. solani*.

The potential of plant extracts in the control of plant pathogens is facilitated by the presence of active ingredients in the form of secondary metabolites. Ferulic acid and Pyroligneous acid are the active ingredients found in *O. sativa* husk and *Q. phillyraeoides* extracts respectively (Yoshida et al. 2000). These compounds are biodegradable, do not incite pathogen resistance and are not toxic to man and livestock. It must be stressed, however, that these active ingredients are not solely responsible for fungitoxicity of the extracts to the test pathogens in this study as revealed in Table 4. The existence of a synergy between the active ingredients and other secondary metabolites such as alkaloids and phenols in plant extracts enhances their potential in plant disease control. Similarly, alkaloids have been implicated in the control of phytopathogenic bacteria (Olugbade and Odebiyi 1992). The ability of the extracts to effectively inhibit the mycelial growth of all the rot-causing fungi *in vitro* is an indication of their broad-spectrum activity.

The success of botanicals in the control of plant pathogens can be determined by the medium of extraction. Some authors have reported that organic solvents such as ethanol extract active ingredients in plant extracts better than water and that they are more effective in controlling plant pathogens (Fuerhake 1984; Lale and Abdulrahaman 1998). However, organic solvents are chemicals and the implication of chemical control in our natural ecosystem cannot be over emphasized. Consequently, aqueous extracts were used in both the *in vitro* and *in vivo* trials in this study which are easy to prepare, cheap and are not toxic. The efficacy of plant extracts is also influenced by the part of the plant that is used for extraction. The active ingredients are often more concentrated in plant storage organs such as roots, tubers, fruits, rhizomes and seeds. Peluola (2005) reported that the concentration of azadirachtin active ingredient was comparatively higher in neem seeds than the leaves or bark.

The presence of secondary metabolites in *O. sativa* and *Q. phillyraeoides* extracts caused the inhibition of radial growth and spore germination of the test pathogens *in vitro*, which confirms the report of previous workers (Dhaliwal et al. 1993; Ejechi and Souzey 1999). The difference recorded in the efficacy of the extracts may be due to the solubility of their active ingredients in water.

The concentration of these extract metabolites is also an important determinant of their ability to inhibit pathogens. Some pathogens have the ability to biodegrade phenols of some botanicals; this reason is responsible for their inability to control such pathogens. *Oryza sativa* husk extract significantly reduced tuber rot *in vivo* at lower concentration as shown in Table 2. These results are consistent with the findings of (Abiala 2008) who reported lower inhibitory concentration ≤ 5% w/v for this extract in the *in vitro* control of *M. fijiensis.*

Injury to yam tubers in the form of wounds enhances the entry of fungal pathogens. This normally occurs during weeding, harvesting, through the feeding lesions of nematodes and insects or while in transit to the market. Rot development was influenced by the degree of injury done to the tubers in this study. Tables 2 and 3 showed that tubers that were bored and inoculated with test pathogens had higher rot incidence than those that were wounded or scratched and inoculated. This result agrees with the findings of (Ogaraku and Usman 2008) who reported a direct relationship between the degree of damage to yam tubers and rot incidence. However, disease incidence and development are primarily facilitated by the presence of a virulent pathogen, susceptible host, and favourable environment (Manjula et al. 2005). There was significant difference (P ≤ 0.05) between the extracts in the control of rot between inoculated tubers that were covered and those that were exposed. They were more effective in controlling rot in tubers that were inoculated and exposed, while rot control was less effective among inoculated tubers that were covered. The higher rot values obtained in replicate tubers that were inoculated and covered could be attributed to the high relative humidity present in the covered treatments, which favours disease development. Conversely, the reduced rot recorded in inoculated and exposed tubers may be attributed to the low relative humidity which discourages fungal infection. Generally, fungal biodeterioration increases at relative humidity greater than 60%, and their virulence diminishes with decreasing relative humidity below this level. These results agree with the findings of Ikotun (1983a) who reported high relative humidity as an important factor in rot initiation of yam tubers.

Post-harvest biodeterioration of ware yam largely starts from the field, resulting from the injuries on yam tubers before storage. The invading fungal pathogens are subsequently carried from the field to the yam barn. In most cases, these infections are latent and only become noticed after incubation and the infected tubers start to rot. However, besides human activities, insects pests, rodents and nematodes that predispose yam tubers to rot, other factors such as sprouting, tuber respiration, temperature and the degree of resistance or susceptibility of yam genotypes to invading pathogens also influence yam storage. Cultural practices such as continuous cropping also encourage the persistence of yam pathogens and enhance their transition from the previous cropping season to the next season. Temperature influences yam storability. Yams store best at 28 ± 2°C in Nigeria. Although storage at lower temperaturs reduces microbial rotting, it causes chilling damage to yam tubers. Storage at temperatures higher than 30°C increases the incidence of xerophilic fungi like *Aspergillus niger* and *Penillium oxalicum*. Therefore, correct manipulation of temperature during storage can also prolong the shelf life of ware yam.

## Conclusion

Tuber wound is the major factor that predisposes pre and post-harvest yam to fungal biodeterioration. The depth of injury to the yam tubers influenced the degree of rot by the test pathogens in this study. Treatments that were bored and inoculated with rot-causing fungi exhibited the highest degree of rot, followed by the wounded and then the scratched tubers. A vital inference and advice to peasant farmers, who are the target beneficiary of this research, therefore, is for them to try as much as possible to avoid or reduce injury to yam tubers during harvest to the barest minimum to prevent losses from yam pathogens. The promising potential of both *O. sativa* and *Q. phillyraeoides* extracts in rot inhibition in this study recommends their development as prospective biopesticide formulation for tuber rot control of white yam.
